# The role of *NKX2-5* gene polymorphisms in congenital heart disease (CHD): a systematic review and meta-analysis

**DOI:** 10.1186/s43044-021-00199-w

**Published:** 2021-08-21

**Authors:** Sana Ashiq, Kanwal Ashiq, Muhammad Farooq Sabar

**Affiliations:** 1 Centre for Applied Molecular Biology, University of the Punjab, 87-West Canal Bank Road, Thokar Niaz Baig, Lahore, 53700 Pakistan; 2 Punjab University College of Pharmacy, University of the Punjab, Lahore, Pakistan; 3 Faculty of Pharmaceutical Sciences, Superior University, Lahore, Pakistan

**Keywords:** *NKX2-5*, Polymorphisms, Congenital heart diseases, Meta-analysis, CHDs

## Abstract

**Background:**

The gene *NKX2-5* is a key transcription factor that plays an essential role in normal cardiac development. Although some recent studies have studied the role of polymorphisms in the *NKX2-5* gene in congenital heart diseases (CHDs), the results were not consistent and remained uncertain. Therefore, we conduct a review of literature and investigate the association of genetic polymorphisms with CHDs.

**Results:**

We selected seventeen studies regarding the association of *NKX2-5* gene rs2277923 polymorphism with CHDs. Overall, in all the tested genetic models, the 63A > G polymorphism was not significantly associated with increased congenital heart defects risk. We used pooled odds ratios (OR) to calculate the association of CHDs with rs2277923 including allelic model: OR 1.00, 95% CI 0.82–1.21; homozygote model: OR 0.95, 95%CI 0.68–1.33, recessive model: OR 0.89 CI 0.70–1.13, heterozygote model: OR: 1.09, 95%CI 0.87–1.37, dominant model: OR 1.08 CI 0.82–1.42 and overdominant model: OR 1.17 CI 1.01–1.35. In addition, our analysis suggests that no publication bias exists in this meta-analysis.

**Conclusions:**

Our findings suggested that 63A > G polymorphism in the *NKX2-5* gene was not significantly associated with congenital heart defects. However, in the future, more studies with increased sample size are required that may provide us more definite conclusions.

**Supplementary Information:**

The online version contains supplementary material available at 10.1186/s43044-021-00199-w.

## Background

Congenital heart diseases (CHDs) or congenital heart defects are defects in great vessels or the heart that arise during cardiac development in the embryo [1, 2]. CHDs are considered one of the major causes of mortality and morbidity in infants. Globally, it is the most common disease with an estimated prevalence of six per thousand live births [3]. Every year, 1.35 million infants are born with these cardiac defects worldwide [4]. The 2020 classification system is being used nowadays to classify congenital heart defect patients into mild, moderate, and severe [5]. It can be further divided as isolated lesion and complex lesion in combination with various heart defects, or it may occur as syndromic CHDs [6]. The patient suffering from CHDs clinically presents with cough, difficulty in breathing, repeated chest infections, and mostly failure to thrive depending upon the type or subcategory of CHD [7]. It is a multifactorial disease that involves genetic, as well as environmental risk factors [8]. The extrinsic factors include abnormal embryonic development due to the lack of essential nutrients or use of an excessive toxic substance such as thalidomide, alcohol, smoking, hypoxia, anticonvulsants, and antidepressants, while the intrinsic factors include maternal diseases such as obesity, gestational diabetes, and maternal rubella virus infections which can cause CHD in 90% of cases [9]. The genetic variants in the genes encoding structural proteins, signal transduction, and transcription factors including T-box factors (*TBX*), *NKX2-5*, and the *GATA* can cause perturbance in normal cardiac development [10]. The *NKX2-5* gene is a key player in almost all phases of cardiac development including septation, regulation of cardiac progenitors cell numbers, valve formation, and conduction system development [11]. The *NKX2-5* contains two exons and situated on chromosome 5q34 that encodes the 324 amino acid protein. The gene belongs to the family of homeodomain-containing transcription factors which interacts with DNA through its helix-turn-helix DNA-binding motif. Thus, a single-nucleotide polymorphism (SNP) can disrupt the gene function resulting in abnormal cardiac morphogenesis [12]. The rs2277923 is a synonymous variant in which arginine is replaced by guanine at position 63 on exon 1. This polymorphism affects the normal gene function that was first reported in 52 control subjects by Benson et al. in 199 [13].

### Rationale

There are many inconsistencies in the published study results regarding the role of rs2277923 in the *NKX2-5* gene, including Iranian [14] Asian [15] Moroccan [16], and Caucasian population [17]; hence, it is needed to analyze all the available published literature that provide us the most definite results for the role of rs2277923 polymorphism in congenital heart diseases.

## Methods

### Aims

The purpose of the present study was to determine the association of rs2277923 polymorphism in the *NKX2 -5* gene with congenital heart defects.

### Design

This study was done according to the standard Preferred Reporting Items for Systematic Reviews and Meta-Analyses (PRISMA) 2009 guidelines, and it is also registered with PROSPERO (PROSPERO registration number CRD42020207952). A PRISMA checklist is also given as a Additional file 1.

### Literature search

Electronic databases of Ovid, PubMed, Web of Science, Cochrane Library, Medline, and EMBASE were searched till March 30, 2021, by using the following keywords and MeSH terms: ‘CHD,’ ‘NKX2-5,’ ‘worldwide,’ ‘congenital heart disease,’ ‘gene polymorphism,’ ‘variant,’ ‘genotype,’ and ‘mutation.’ Further identification of each potentially eligible article was conducted based on a manual search of the individual article reference list. In the final analysis, all the duplicate research articles were not included.

### The characteristics of participants

For the selection of studies, the following criteria were used (1) The full-length published research studies that investigated the relationship of *NKX2-5* gene polymorphism (rs2277923) with congenital heart diseases (2) The retrospective case–control studies (3) Adequate data were available for the genetic statistical analysis. The review articles either narrative or systematic, meta-analysis, doctoral thesis, not published in the English language, not designed as a case–control study, or research articles that not provided enough data for the statistical analysis were excluded from the present study.

## Process

### Data extraction

A predesigned data extraction table was used to minimize the selection bias. All three authors individually assessed and extracted the required detailed information from each included study. The following details were abstracted from each selected article: author names, country, publication year, the sample size of both case and control subjects, baseline characteristics, genotypic method, distribution of alleles and genotypes, and evidence confirming the Hardy–Weinberg equilibrium (HWE). Each study’s detailed characteristics are also summarized in Tables 1 and 2.

**Table 1:** Characteristics of each study included in the final analysis

Serial no	Authors	Year	Country	Gender wise distribution (M/F)	SOC	Sample size		Genotyping	NOS score
						Cases (N)	Controls (N)		
1	Behiry	2019	Egypt	44% = M 56% = F	PB	150	90	PCR-Sequencing	8
2	Peng	2010	China	NA	PB	135	114	PCR-Sequencing	7
3	Cao	2015	China	29 = M 41 = F	HB	70	136	PCR-Sequencing	7
4	Ketharnathan	2015	India	26 = M 24 = F	PB	50	50	PCR-Sequencing/RT-PCR	8
5	Pang	2012	China	110 = M 103 = F	HB	213	194	PCR-Sequencing	8
6	Dinesh	2010	India	NA	PB	150	70	PCR-Sequencing	7
7	Ouyang	2011	China	NA	PB	125	105	PCR-Sequencing	7
8	Wang et al.	2019	China	157 = M 282 = F	PB	439	567	Multiplex PCR, qPCR, Sequencing	8
9	Liu [1]	2009	China	NA	PB	160	200	PCR-Sequencing	7
10	Liu [2]	2009	China	NA	PB	180	200	PCR-Sequencing	7
11	Han	2011	China	NA		81	52		7
12	Xie	2013	China	71 = M 65 = F	PB	136	200	PCR-Sequencing	
13	Xiong	2013	China	166 = M 58 = F	PB	224	121	PCR, DHPLC-Sequencing	7
14	Yin	2018	China	59 = M 39 = F	PB	98	200	PCR-direct sequence analysis	8
15	Zhang	2009	China	132 = M 98 = F	PB	230	200	PCR, denaturing high-performance liquid chromatography, and sequencing	8
16	Zhao	2020	China	NA	HB	620	620	PCR, MassARRAY system	8
17	Shi	2005	China	NA	NA	110	110	––	8

**Table 2:** The allele frequencies and genotypes distributions of *NKX2-5* polymorphism rs2277923

Serial no	Authors	Year	Genotypes distribution						Alleles distribution			
			Cases			Controls			Cases		Controls	
			AA	AG	GG	AA	AG	GG	A	G	A	G
1	Behiry	2019	09	87	54	0	32	58	105	195	32	148
2	Peng	2010	-	-	-	-	-	-	22.2	77.8	22.8	77.2
3	Cao	2015	20	37	13	24	65	47	77	63	113	159
4	Ketharnathan	2015	20	19	11	43	7	0	59	41	93	7
5	Pang	2012	27	100	86	29	80	85	36.2	63.8	35.6	64.4
6	Dinesh	2010	77	49	24	34	25	11	67.7	32.3	66.4	33.6
7	Ouyang	2011	-	-	-	-	-	-	46.1	53.9	36.7	63.3
8	Wang et al.	2019	–	–	–	–	–	–	87	352	86	481
9	Liu [1]	2009	30	70	60	48	94	58	40.6	59.4	47.5	52.5
10	Liu [2]	2009	32	85	63	48	94	58	41.4	58.6	47.5	52.5
11	Han	2011	08	43	30	04	25	23	36.4	63.6	31.7	68.3
12	Xie	2013	–	–	–	–	–	–	75.4	24.6	76.2	23.8
13	Xiong	2013	–	–	–	–	–	–	79.2	20.8	82.3	17.7
14	Yin	2018	11.2	50	38.8	18.5	49	32.5	36.2	63.8	43	57
15	Zhang	2009	30	107	93	26	98	76	36.3	63.7	37.5	62.5
16	Zhao	2020	93	310	217	75	254	291	40	60	32.6	67.4
17	Shi	2005	7	27	76	07	33	70	19	81	37.7	62.3

### Quality evaluation

Each author evaluated the quality of published studies, and any discrepancies were solved by discussion to achieve a consensus. According to the Newcastle–Ottawa Scale (NOS), the quality of each original study was evaluated. The Newcastle–Ottawa Scale ranges from Worst (0) to Best (9).

### Statistical analysis

The role of *NKX2-5* polymorphism (rs2277923) in the pathogenesis of congenital heart disease was checked by calculating the pooled odds ratio (ORs) and 95% confidence interval (CI). The chi-squared test and I^2^ statistic were used to calculate the heterogeneity between each included study. In the presence of significant heterogeneity, we used a random-effects model (DerSimonian–Laird). While in the absence of heterogeneity, a fixed-effect model (Mantel–Haenszel) was used. The genetic models used for rs2277923 were allelic model, heterozygote and homozygote model, overdominant, dominant, and recessive model. To check the stability of the results, we performed the sensitivity test. Publication bias was assessed with the funnel plot. We performed the Begg’s and Egger’s test to evaluate the publication bias, and publication bias was considered present when *p* ≤ 0.05. The MetaGenyo tool was used for performing the meta-analysis.

## Results

### Characteristics of final included studies

**Fig. 1:**
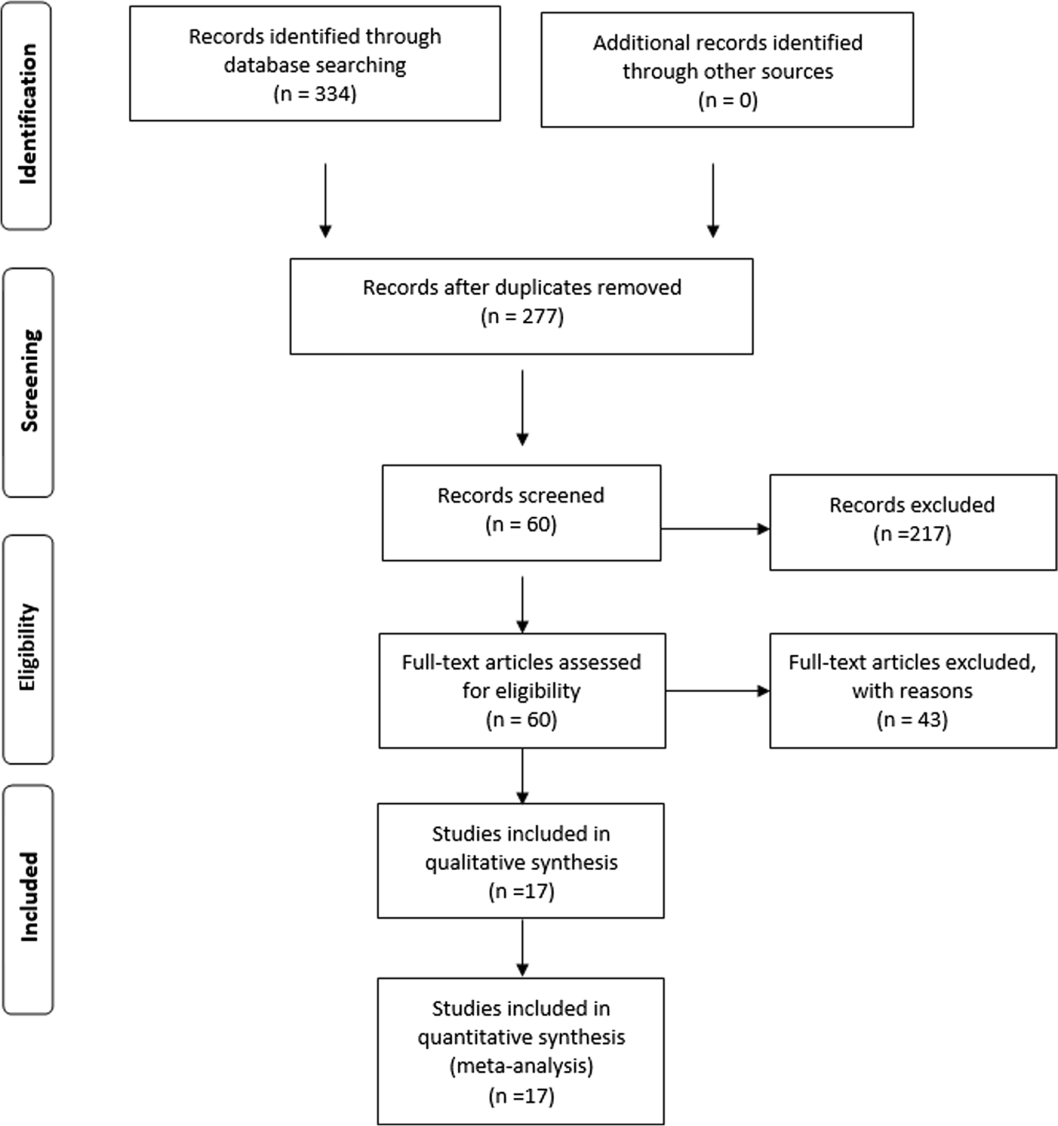
Flow diagram of study selection

Initially, 334 published studies were selected, of which 217 articles were not included as they did not study the rs2277923 association with congenital heart diseases, thus 60 articles were selected for further evaluation. Among these, 17 original articles met our inclusion criteria; therefore, these were selected further for final analysis. The complete screening method for the literature search is given in Fig. 1. Of these seventeen studies, one study included the Egyptian population, fourteen studies included the Asian population, whereas two studies were performed in Caucasian ethnicity. The controls of three included studies were based on hospital-based population (HB), and the other fourteen were from the general population-based (PB) [11, 12, 15, 18–31]. The quality of all included studies was ranged from 7–8. The detailed baseline characteristics are explained in Table 1. The controls included in seventeen studies were following the Hardy–Weinberg equilibrium. The allele frequencies and genotype distribution are given in Table 2.

### Association of rs2277923 with congenital heart diseases

**Fig. 2:**
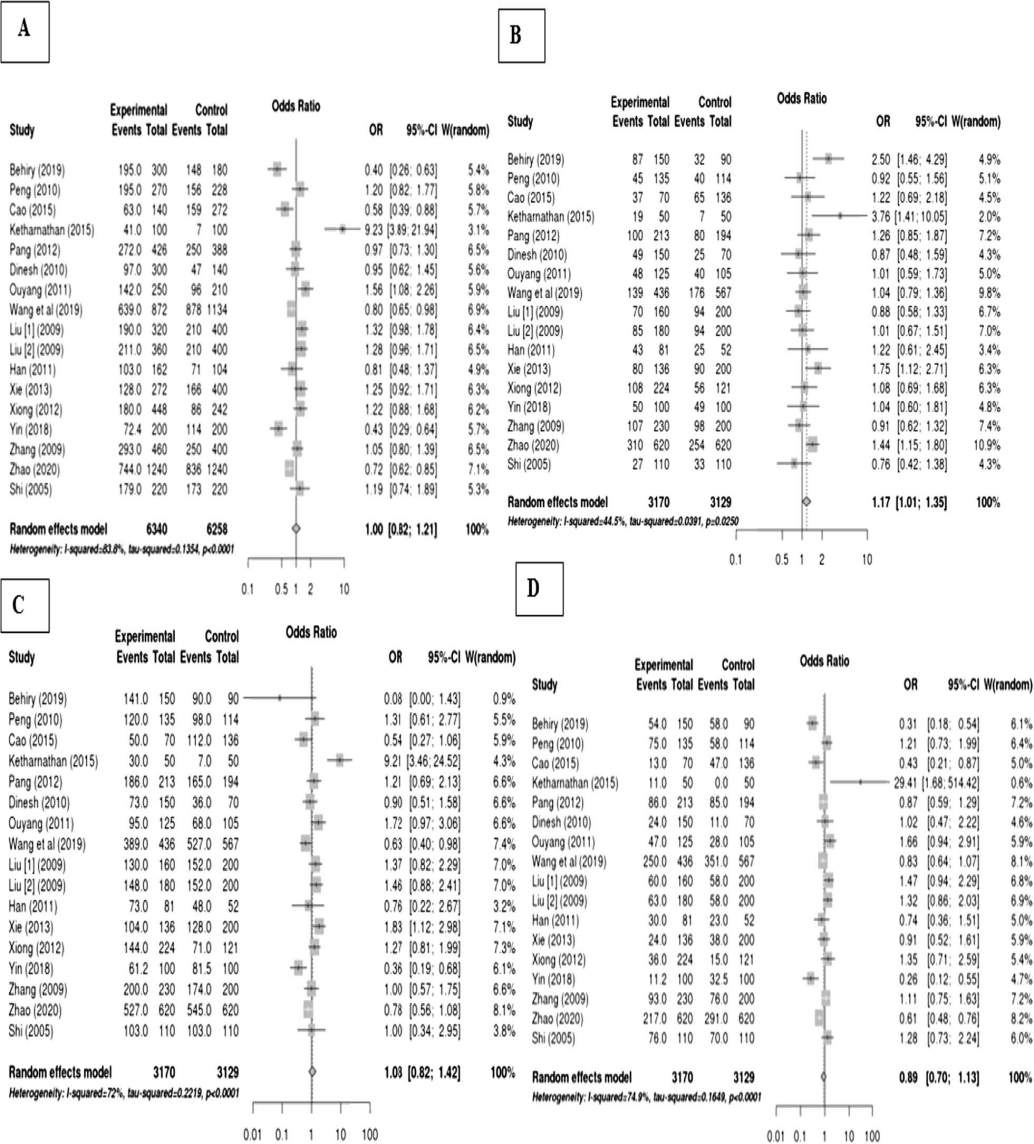
Final data analysis for rs2277923 association with congenital heart defects and the forest plot for different genetic models showing results as **a** allelic, **b** overdominant, **c** dominant, and **d** recessive, respectively

**Fig. 3:**
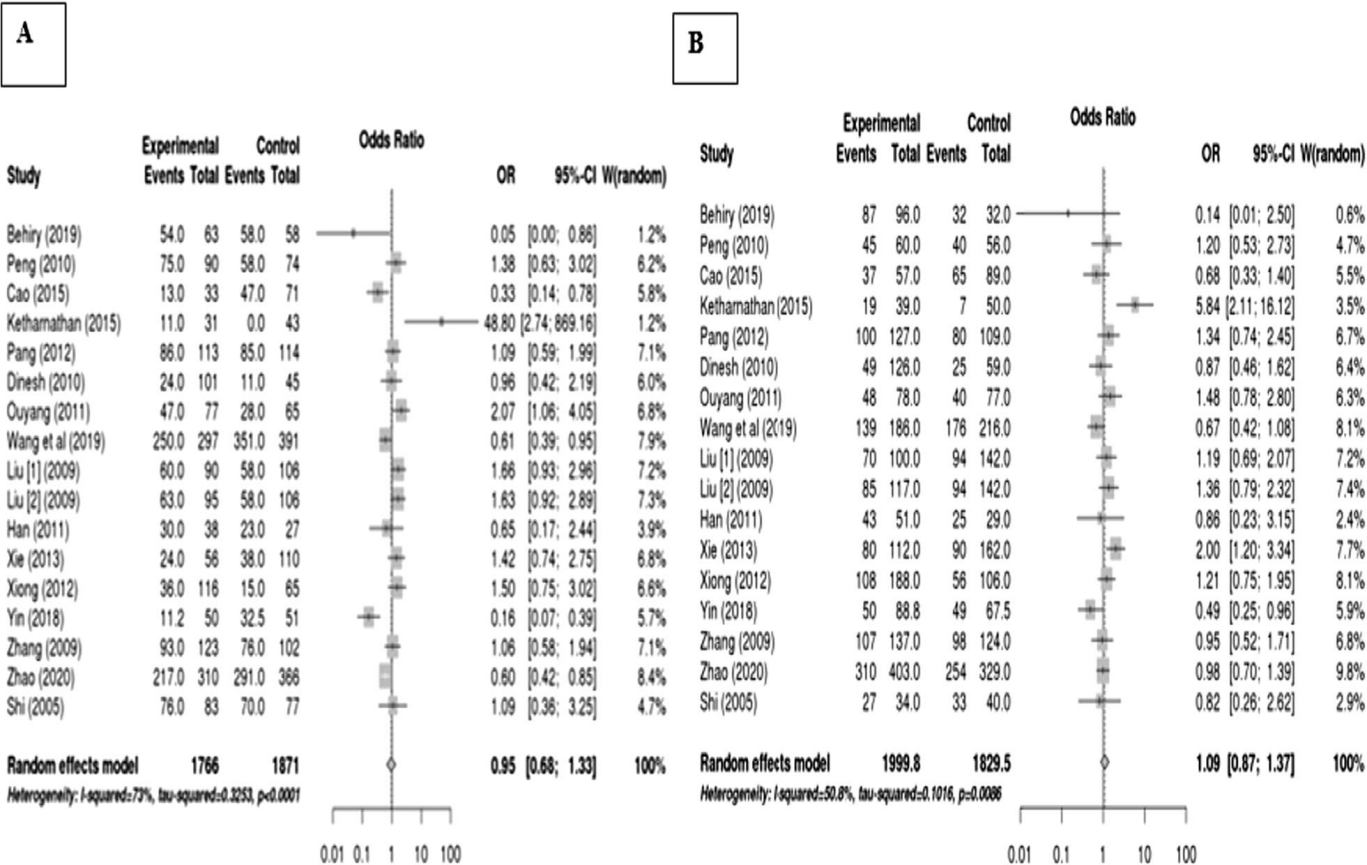
Final analysis for rs2277923 association with congenital heart diseases A: homozygote forest plot and B: heterozygote forest plot

The pooled results of rs2277923 in the *NKX2-5* gene show a non-significant association with CHDs. We selected the random-effects models to combine all information. Overall, the rs2277923 SNP not increased the risk of congenital heart diseases in each tested genetic model (allelic model: OR 1.00, 95% CI 0.82–1.21; overdominant model: OR 1.17 CI 1.01–1.35; dominant model: OR 1.08 CI 0.82–1.42; recessive model: OR 0.89 CI 0.70–1.13). The meta-analysis of these four models is shown in Fig. 2. The results of homozygote and heterozygote models were given as, respectively: OR 0.95, 95%CI 0.68–1.33 and OR: 1.09, 95%CI 0.87–1.37 (Fig. 3).

### Sensitivity analysis

After sequentially excluding each study, the overall changes in OR with a 95% confidence interval were not statistically significant, suggesting the reliability and stability of current meta-analysis results.

### Publication bias

**Fig. 4:**
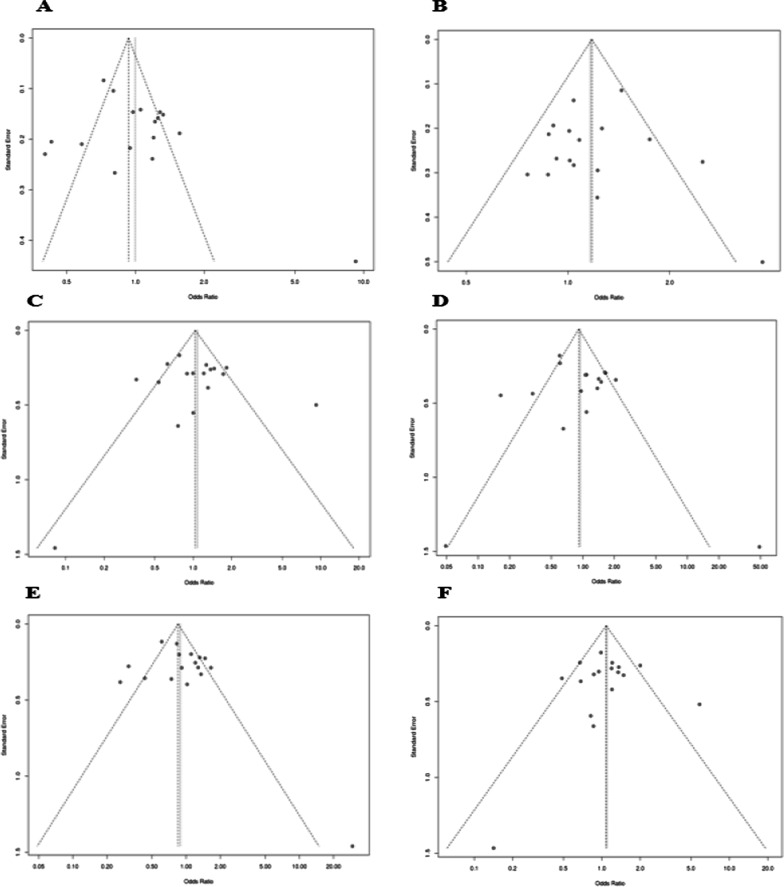
Final publication bias analysis for rs2277923 relationship with CHDs and the funnel plot for different genetic models showing results as **a** allelic, **b** overdominant, **c** dominant, **d** recessive, **e** homozygote, and **f** heterozygote

In the current study, funnel plot analysis does not explain any apparent asymmetry in each tested genetic model as illustrated in Fig. 4. Moreover, the Eggers test also confirmed no statistically significant effect. For the rs2277923, the *p* value for each genetic model was given as: allelic: 0.19, overdominant: 0.96, dominant: 0.82, recessive: 0.32, homozygote: 0.66 and heterozygote: 0.89.

## Discussion

To date, few systematic reviews and meta-analyses have been done to find the association of rs2277923 association with congenital heart defects [13, 32], but the present analysis was the most comprehensive assessment of rs2277923 role in CHDs. In addition, our analysis includes recent studies conducted (till 2020). The *NKX2-5* gene is the vital gene that interacts with other transcriptional factors, including the T-box transcription factor (*TBX5*) and *GATA4*; thus, it plays a crucial role in cardiac development. Hence, a single-nucleotide polymorphism can alter the gene function that ultimately affects the growth and heart structural morphogenesis [33].

In the current study, we analyzed all the available literature on rs2277923 polymorphism and congenital heart defects, and the pooled results suggest a non-significant relationship between congenital heart diseases and the selected polymorphism. Our analysis showed that in different ethnic groups, minor allele was not associated with CHDs.

Our results are in accordance with those performed by the Kalayinia et al. as they showed the overall percentage of the mutant and wild allele was 2.5%, and 65.8%, respectively [14], but in contrast to those reported by the Cao et al., they found that genotypic frequency distribution significantly differed between the control group and patients with atrial septal defects as *p* = 0.009 [34]. Xie et al. also conducted a meta-analysis of thirteen original studies and reported the non-significant association (*p* = 0.39, OR = 1.10, 95% CI = 0.88–1.38) [32]. Similarly, Liang et al. meta-analysis results are consistent with our findings. They include eight studies for final analysis and reported *p* = 0.73 for the allelic model [35]. Similar conclusions were reported in Caucasians [17]. Wang et al. revealed a significant association for rs2277923 with CHDs in the Chinese population [13]. We did the Begg and Egger test on all included studies so that any false-positive result due to publication bias can be eliminated in the current analysis [36]. We found no publication bias in the current meta-analysis, which further increases the reliability of our included studies.

## Limitations

Although the cumulative results of this study are quite comprehensive, however, certain limitations also exist in this meta-analysis. First, we only chose one single-nucleotide polymorphism of the selected gene that may be influenced by gene–environment and gene–gene interactions. Second, we cannot exclude the possibility of publication bias because, in this study, we selected only English language published literature.

## Conclusions

We concluded that *NKX2-5* rs2277923 single-nucleotide polymorphism was not significantly associated with congenital heart defects. It is suggested that there is a need for further meta-analysis with a larger cohort size in various subgroups that may provide us the more definite conclusions. Moreover, in the future, more genetic variants should be included in the analysis that may help us in better understanding the genetic mechanism involved in the pathogenesis of CHDs.

## Supplementary Information

**Additional file 1.** PRISMA 2009 Checklist.

## Data Availability

Provided as supplementary files.

## Abbreviations

CHDs: Congenital heart diseases
TBX5: T-box transcription factors
SNP: Single-nucleotide polymorphism
PRISMA: Preferred reporting items for systematic reviews and meta-analyses
HWE: Hardy–Weinberg equilibrium
NOS: Newcastle–Ottawa scale
CI: Confidence interval
OR: Odds ratio
HB: Hospital based
PB: Population based

## References

[1] Bouma BJ, Mulder BJ (2017). Changing landscape of congenital heart disease. Circ Res.

[2] Shabana NA, Shahid SU, Irfan U (2020). Genetic contribution to congenital heart disease (CHD). Pediatr Cardiol.

[3] Muntean I, Togănel R, Benedek T (2017). Genetics of congenital heart disease: past and present. Biochem Genet.

[4] Ashiq S, Ashiq K (2020). Genetic perspective of the congenital heart disease. Pak Heart J.

[5] Baumgartner H, De Backer J, Babu-Narayan SV, Budts W, Chessa M, Diller GP, Lung B, Kluin J, Lang IM, Meijboom F, Moons P (2021). 2020 ESC guidelines for the management of adult congenital heart disease: The task force for the management of adult congenital heart disease of the European Society of Cardiology (ESC). Endorsed by: Association for European Paediatric and Congenital Cardiology (AEPC), International Society for Adult Congenital Heart Disease (ISACHD). Eur Heart J.

[6] Pate N, Jawed S, Nigar N, Junaid F, Wadood AA, Abdullah F (2016). Frequency and pattern of congenital heart defects in a tertiary care cardiac hospital of Karachi. Pak J Med Sci.

[7] Suluba E, Shuwei L, Xia Q, Mwanga A (2020). Congenital heart diseases: genetics, non-inherited risk factors, and signaling pathways. Egypt J Med Hum Genet.

[8] Peng J, Meng Z, Zhou S, Zhou Y, Wu Y, Wang Q, Wang J, Sun K (2019). The non-genetic paternal factors for congenital heart defects: a systematic review and meta-analysis. Clin Cardiol.

[9] Kalisch-Smith JI, Ved N, Sparrow DB (2020). Environmental risk factors for congenital heart disease. Cold Spring Harb Perspect Biol.

[10] Su W, Zhu P, Wang R, Wu Q, Wang M, Zhang X, Mei L, Tang J, Kumar M, Wang X, Su L (2017). Congenital heart diseases and their association with the variant distribution features on susceptibility genes. Clin Genet.

[11] Behiry EG, Al-Azzouny MA, Sabry D, Behairy OG, Salem NE (2019). Association of NKX2-5, GATA4, and TBX5 polymorphisms with congenital heart disease in Egyptian children. Mol Genet Genom Med.

[12] Peng T, Wang L, Zhou SF, Li X (2010). Mutations of the GATA4 and NKX2. 5 genes in Chinese pediatric patients with non-familial congenital heart disease. Genetica.

[13] Wang Z, Zou L, Zhong R, Zhu B, Chen W, Shen N, Ke J, Lou J, Song R, Miao XP (2013). Associations between two genetic variants in NKX2-5 and risk of congenital heart disease in Chinese population: a meta-analysis. PLoS ONE.

[14] Kalayinia S, Biglari A, Rokni-Zadeh H, Mahdavi M, Rabbani B, Maleki M, Mahdieh N (2018). The Nkx2-5 gene mutations related to congenital heart diseases in Iranian patients population. Int Cardiovasc Res J.

[15] Dinesh SM, Kusuma L, Smitha R, Savitha MR, Krishnamurthy B, Narayanappa D, Ramachandra NB (2010). Single-nucleotide polymorphisms of NKX2. 5 found in congenital heart disease patients of Mysore. South India. Genet Test Mol Biomark.

[16] El Bouchikhi I, Bouguenouch L, Moufid FZ, Houssaini MI, Belhassan K, Samri I, Joutei AT, Ouldim K, Atmani S (2017). NKX2-5 molecular screening and assessment of variant rate and risk factors of secundum atrial septal defect in a Moroccan population. Anatol J Cardiol.

[17] Alcántara-Ortigoza MA, De Rubens-Figueroa J, Reyna-Fabian ME, Estandía-Ortega B, González-del Angel A, Molina-Álvarez B, Velázquez-Aragón JA, Villagómez-Martínez S, Pereira-López GI, Cruz-Martínez V, Álvarez-Gómez RM (2015). Germline mutations in NKX2-5, GATA4, and CRELD1 are rare in a Mexican sample of Down syndrome patients with endocardial cushion and septal heart defects. Pediatr Cardiol.

[18] Cao Y, Lan W, Li Y, Wei C, Zou H, Jiang L (2015). Single nucleotide polymorphism of NKX2-5 gene with sporadic congenital heart disease in Chinese Bai population. Int J Clin Exp Pathol.

[19] Ketharnathan S, Koshy T, Sethuratnam R, Paul S, Venkatesan V (2015). Investigation of NKX2. 5 gene mutations in congenital heart defects in an Indian population. Genet Test Mol Biomark.

[20] Pang S, Shan J, Qiao Y, Ma L, Qin X, Wanyan H, Xing Q, Wu G, Yan B (2012). Genetic and functional analysis of the NKX2-5 gene promoter in patients with ventricular septal defects. Pediatr Cardiol.

[21] Ouyang P, Saarel E, Bai Y, Luo C, Lv Q, Xu Y, Wang F, Fan C, Younoszai A, Chen Q, Tu X (2011). A de novo mutation in NKX2. 5 associated with atrial septal defects, ventricular noncompaction, syncope and sudden death. Clin Chim Acta.

[22] Wang H, Liu Y, Li Y, Wang W, Li L, Meng M, Xie Y, Zhang Y, Zi Y, Han S, Zeng J (2019). Analysis of NKX2-5 in 439 Chinese patients with sporadic atrial septal defect. Med Sci Monit.

[23] Liu XY, Yang YQ, Yang Y, Lin XP, Chen YH (2009). Mutation of NKX2-5 gene in patients with atrial septal defect. Chin J Pediatr.

[24] Liu XY, Yang YQ, Yang Y, Lin XP, Chen YH (2009). Novel NKX2-5 mutations identified in patients with congenital ventricular septal defects. Chin Med J.

[25] Han Z, Tang Y, Chen Y, Hu D (2011). Mutation screening of NKX2. 5 gene and its relationship to simple congenital heart disease in 81 patients. Chin Circ J.

[26] Xie WH, Chang C, Xu YJ, Li RG, Qu XK, Fang WY, Liu X, Yang YQ (2013). Prevalence and spectrum of Nkx2. 5 mutations associated with idiopathic atrial fibrillation. Clinics.

[27] Xiong F, Li Q, Zhang C, Chen Y, Li P, Wei X, Li Q, Zhou W, Li L, Shang X, Xu X (2013). Analyses of GATA4, NKX2. 5, and TFAP2B genes in subjects from southern China with sporadic congenital heart disease. Cardiovasc Pathol.

[28] Yin J, Qian J, Dai G, Wang C, Qin Y, Xu T, Li Z, Zhang H, Yang S (2019). Search of somatic mutations of NKX2-5 and GATA4 genes in Chinese patients with sporadic congenital heart disease. Pediatr Cardiol.

[29] Zhang W, Li X, Shen A, Jiao W, Guan X, Li Z (2009). Screening NXK2. 5 mutation in a sample of 230 Han Chinese children with congenital heart diseases. Genet Test Mol Biomark.

[30] Zhao M, Diao J, Huang P, Li J, Li Y, Yang Y, Luo L, Zhang S, Chen L, Wang T, Zhu P (2020). Association of maternal diabetes mellitus and polymorphisms of the NKX2.5 gene in children with congenital heart disease: a single centre-based case-control study. J Diabetes Res.

[31] Shi L, Shen AD, Li XF, Bai S, Guan XL, Li ZZ (2005). Mutation screening of Nkx2. 5 gene and associated study in Exon1 in Chinese with congenital heart disease. J Capital Univ Med Sci.

[32] Xie X, Shi X, Xun X, Rao L (2016). Associations of NKX2-5 genetic polymorphisms with the risk of congenital heart disease: a meta-analysis. Pediatr Cardiol.

[33] Mattapally S, Singh M, Murthy KS, Asthana S, Banerjee SK (2018). Computational modeling suggests impaired interactions between NKX2. 5 and GATA4 in individuals carrying a novel pathogenic D16N NKX2. 5 mutation. Oncotarget.

[34] Cao Y, Wang J, Wei C, Hou Z, Li Y, Zou H, Meng M, Wang W, Jiang L (2016). Genetic variations of NKX2-5 in sporadic atrial septal defect and ventricular septal defect in Chinese Yunnan population. Gene.

[35] Liang B, Jing M, Liao H (2018). Meta-analysis of NKX 2-5 polymorphisms on the risk of cardiovascular disease. Life Res.

[36] Egger M, Smith GD, Schneider M, Minder C (1997). Bias in meta-analysis detected by a simple, graphical test. BMJ.

